# Retinal and choroidal microvascular characterization and density changes in different stages of diabetic retinopathy eyes

**DOI:** 10.3389/fmed.2023.1186098

**Published:** 2023-07-25

**Authors:** Hui Wang, Xuhui Liu, Xiaofeng Hu, Hua Xin, Han Bao, Shuo Yang

**Affiliations:** ^1^Department of Ophthalmology, Beijing Chaoyang Hospital, Capital Medical University, Beijing, China; ^2^Luoyang Shenzhou Eye Hospital, Henan, China

**Keywords:** diabetic retinopathy, optical coherence tomography angiography, vessel density, perfusion density, superficial capillary plexus

## Abstract

**Background:**

The purpose of this study was to evaluate the changes in fundus vascular density and micromorphological structure of all vascular plexuses during the different stages of diabetic retinopathy (DR), and the correlation between fundus blood flow and the DR severity.

**Methods:**

This observational cross-sectional study was conducted of 50 eyes with different stages of DR, 25 diabetes mellitus (DM) patients without clinical signs of DR and 41 healthy eyes. The foveal avascular zone (FAZ), vessel density of superficial capillary plexus (SCP), and deep retinal capillary plexus (DCP) were acquired by RTVue XR Avanti OCTA device. The perfusion density (PD), skeleton vessel density (SVD) was manually calculated using ImageJ. The area under receiver operating characteristic (ROC) curve was used to determine the diagnostic value of OCTA parameters in distinguishing DR and healthy eyes.

**Results:**

The choroidal VD were significantly higher in the healthy group than in the DM without DR, NPDR, and PDR groups (*p* < 0.001). The mean retinal parafovea VD, PD, and retinal SVD were higher in healthy and DM without DR eyes compared with NPDR and DR eyes in all vascular layers (*p* < 0.001). The parafoveal VD of SCP, and DCP decreased, and FAZ area increased with the exacerbation of DR. The OCTA parameters, including FAZ area, parafovea VD, PD, and SVD in all vascular layers showed significant correlation with DR severity (all *p* < 0.001). ROC curves of OCTA parameters (FAZ area, retinal parafovea VD, retinal PD, and SVD in all vascular layers) for had high sensitivity and specificity in distinguishing DR versus healthy eyes.

**Conclusion:**

The choroidal parafovea VD, retinal parafovea VD, retinal PD, and SVD in the two plexuses decreased, and retinal FAZ area increased significantly with worsening DR. VD, PD, and SVD might be potential early biomarkers indicating the progression of DR before appearance of clinically PDR in patients with DM. In this study, OCTA parameters had high sensitivity and specificity in distinguishing DR and healthy eyes.

## Background

Diabetic retinopathy (DR) is one of the leading causes of blindness worldwide (1). The persistent hyperglycemic state may lead to increased hypoxia and oxidative stress, resulting in systemic microvascular abnormalities (2). Detecting the status and flow changes of these micro vessels at different stages of DR may provide meaningful retinal perfusion status to the ophthalmologists. Previous studies of DR have mostly used fluorescent angiography (FFA) to detect macular capillary abnormalities and suggested that foveal microvascular abnormalities may be useful in assessing DR progression (3). Due to its advantages of non-invasive and high resolution, optical coherence tomography angiography (OCTA) has been more widely used in vessel density evaluation in recent years. OCTA is a new retinal blood flow detection method in recent years, which can non-invasively identify different vascular plexuses of retina and choroidal blood perfusion. OCTA was recently used for the staging of DR, which has acceptable sensitivity and specificity for diagnosis and classification of DR (4).

Previous studies have evaluated the correlation between changes in vascular density (VD) and DR progression by using OCTA techniques (5–9). Recently, some new parameters describing the morphology of fundus microvascular have also been proposed and applied to the evaluation of retinal blood vessels in varies fundus diseases. The fractal dimension (FD) is a quantitative parameter used to describe the complexity of blood vessels, Reif et al. (10) first used OCTA to explore FD when studying the distribution of the mouse ear vascular system. In recent years, it has been widely used to evaluate the pathological features of micro vessels in OCTA imaging (6, 11). Yuan et al. (12) found lower peripapillary VD and peripapillary vessel length density (VLD) were associated with 2 years incident DR. Kim et al. (6) quantified retinal vascular density in healthy and diabetic subjects using retinal OCTA imaging and calculated FD and vascular density index (VDI) after OCTA images processing. Zang et al. (13) evaluated a deep-learning-aided DR classification framework using volumetric OCT and OCTA, which can provide specialist-level DR classification using only a single imaging modality.

Previous studies also showed that OCTA can distinguish between healthy eyes and DR eyes (5, 6, 14, 15).

These previous studies suggested that analysis of retinal blood flow density and microstructural changes by some image processing may be useful in assessing the severity of DR. The aim of this study was to evaluate the changes in fundus vascular density and micromorphological structure of all vascular plexuses during the different stages of diabetic retinopathy (DR), found the correlation between fundus blood flow and the DR severity, and to assesses the ability to use fundus microvascular features to distinguish healthy eyes from those DR eyes.

## Materials and methods

### Study population

The observational cross-sectional study was conducted by following the principles outlined in the Declaration of Helsinki and approved by the Ethics Committee of Beijing Chaoyang Hospital, Capital Medical University. Written informed consent was obtained from the subjects involved with the study. In this study, 144 subjects (116 eyes) were included for evaluating the morphological characteristics of peripapillary and macular region, 28 were excluded due to SSI <55, leaving 41 healthy control eyes, 25 DM patients without DR (no DR group), 27 NPDR eyes (NPDR group) and 23 eyes with PDR (PDR group). The severity of the diabetic eye disease was graded according to the Early Treatment Diabetic Retinopathy Study (ETDRS) classification (16).

The exclusion criteria were: (1) eyes with other types of retinal diseases, including bilateral pathological myopia, optic neuropathy, ophthalmic ischemia syndrome, various types of glaucoma, etc.; (2) previous eye surgery including fundus laser, vitrectomy, intraocular surgery (including cataract surgery), etc.; (3) obscuration of retinal and choroidal images by thick subfoveal hemorrhage or macular disease; (4) any factor that causes significant artifact components in OCTA images that affected the impact on OCTA image quality and caused quantitative analysis errors, such as severe lens opacity, vitreous opacity, etc.; poor image quality from excessive eye motion; images with wrong segmentation of the large vessels, macular deviation from center were excluded; OCTA images with a signal strength index (SSI) <55 were also excluded.

All enrolled patients were acquired of a questionnaire and underwent a detailed ophthalmic examination: best-corrected visual acuity (BCVA), intraocular pressure (IOP), slit-lamp biomicroscopy, dilated fundal examination. Snellen visual acuities were converted to logMAR equivalent for statistical analysis.

### OCTA examinations

OCTA imaging was performed using the RTVue-XR Avanti OCTA system (Optovue, Inc., Fremont, CA, United States). If the image signal strength index (SSI) was less than 55 for the AngioVue, imaging technicians were instructed to obtain retakes, and the scan with the highest quality was included in the analysis. Those images with a signal strength index (SSI) <55 were excluded. Optic nerve fiber layer (ONFL) thickness, retinal thickness (RT) and subfovea choroid thickness (SFCT) were automatically measured by the OCTA built-in measurement tool.

The RTVue-XR Avanti OCTA System (Optovue, Inc., Fremont, CA, United States) system uses the split-spectrum amplitude-decorrelation angiography (SSADA) algorithm (AngioVue Analytics; version 2016.1.0.26; Optovue, Inc.). This device has an A-scan rate of 70,000 scans/s using a commercial spectral-domain OCT system with a center wavelength of 840 nm, a full-width half maximum bandwidth of 45 nm, and an axial scan rate of 70 kHz. Two consecutive volumetric scans, each containing 304 B-scans, were captured in a 3 × 3 mm^2^ scanning area centered on the fovea. In the fast transverse scanning direction, 304 axial scans were sampled to obtain a single 3 mm B-scan. Two repeated B-scans were captured at a fixed position. The OCTA built-in automatic segmentation algorithm automatically separates the boundaries of retinal superficial capillary plexus (SCP) and deep retinal capillary plexus (DCP).

Retinal SCP represents 70% of the inner retina, and DCP represents the outer 30% part of the retina. In brief, the inner boundary of for the SCP was segmented with an inner boundary of 3 μm below the internal limiting membrane (ILM), and the outer boundary was set at 15 μm beneath the inner plexiform layer (IPL). The DCP layer was segmented from 15 to 70 μm posterior to the IPL (14).

#### Vessel density (VD, %)

**Figure 1:**
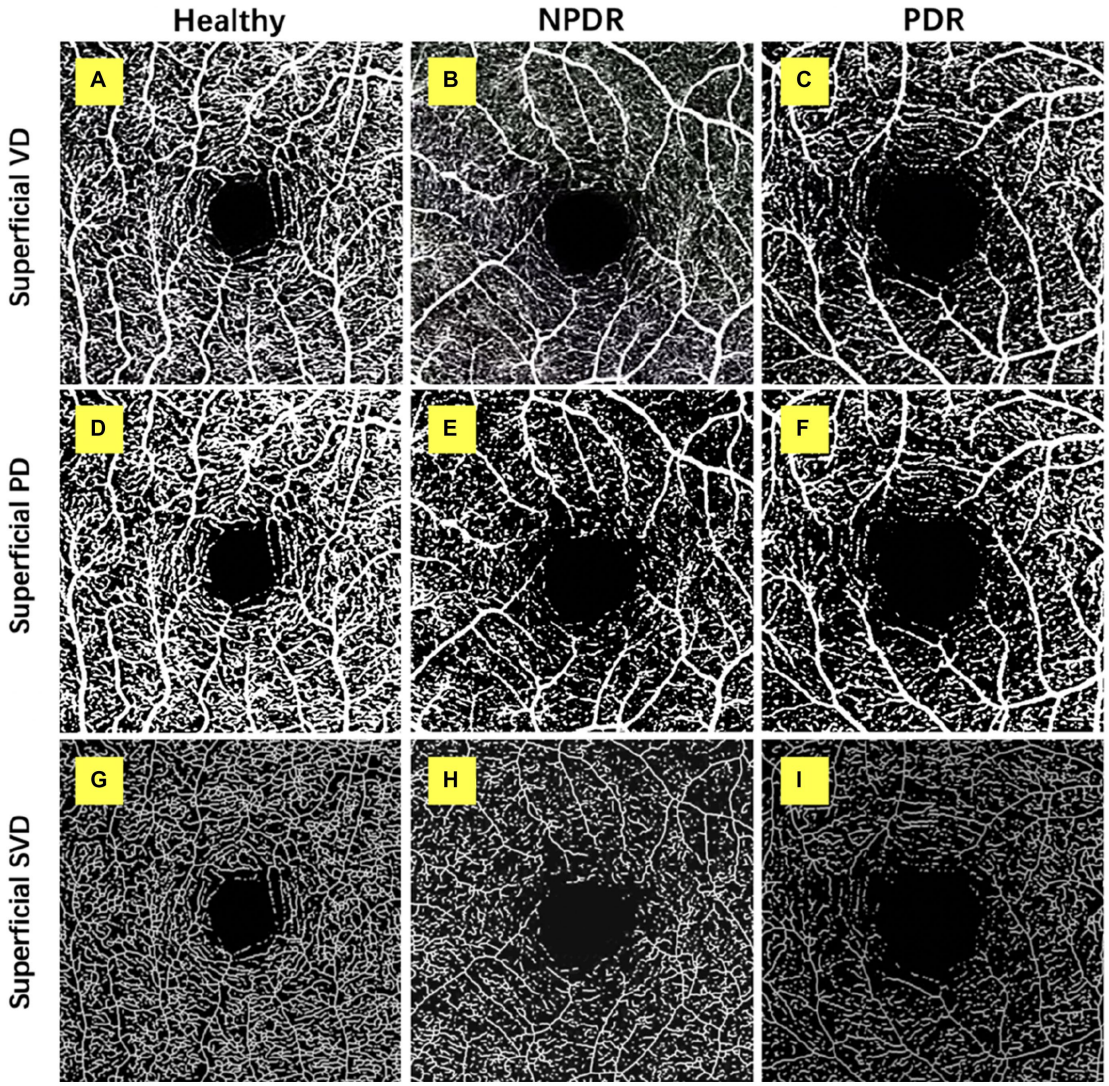
Retinal superficial capillary plexus vascular density (VD), perfusion density (PD), and skeleton vessel density (SVD) in healthy, non-proliferative diabetic retinopathy (NPDR) and diabetic retinopathy (DR) group. Optical coherence tomography angiography (OCTA) images with quantitative image outputs of representative subjects in 3 × 3 mm^2^ areas around the fovea. The OCTA built-in automatic segmentation algorithm automatically separates the boundaries of retinal superficial capillary plexus (SCP). **(A–D)** *En face* representations of retinal perfusion can be viewed as grayscale original, non-segmented OCTA images of retinal vasculature, with noise thresholding. VD automatically measured by the OCTA built-in measurement tool. **(D–F)** A contrast-enhanced binarized image was obtained by using combined adaptive threshold and Hessian filter. This image was used for quantification of PD. **(G–I)** A skeletonized image was obtained by iteratively deleting the pixels in the outer boundary of the binarized image until 1 pixel remained along the width direction of the vessels. This image was used for calculation of SVD.

**Figure 2:**
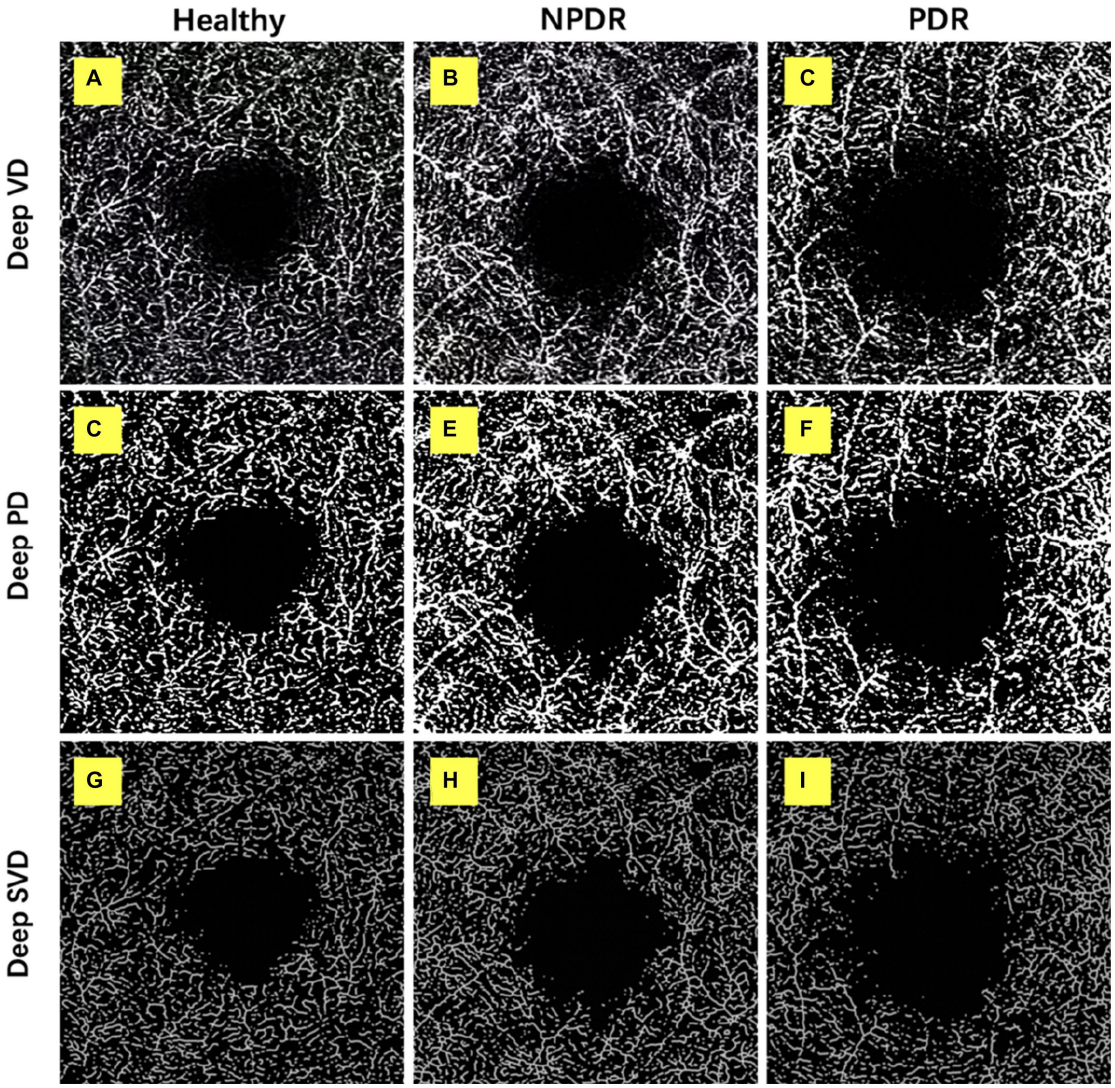
Retinal deep capillary plexus VD, PD, and SVD in healthy, NPDR and DR group. OCTA images with quantitative image outputs of representative subjects in 3 × 3 mm^2^ areas around the fovea. The OCTA built-in automatic segmentation algorithm automatically separates the boundaries of retinal deep capillary plexus (DCP). **(A–D)** *En face* representations of retinal perfusion can be viewed as grayscale original, non-segmented OCTA images of retinal vasculature, with noise thresholding. VD automatically measured by the OCTA built-in measurement tool. **(D–F)** A contrast-enhanced binarized image was obtained by using combined adaptive threshold and Hessian filter. This image was used for quantification of PD. **(G–I)** A skeletonized image was obtained by iteratively deleting the pixels in the outer boundary of the binarized image until 1 pixel remained along the width direction of the vessels. This image was used for calculation of SVD.

The built-in AngioVue Analytics software was used to obtain SCP and DCP blood VD (Figures 1, 2). Only the parafovea (the area centered on the central fovea of the macula, with inner and outer rings 1 mm and 3 mm in diameter, respectively) VD values were analyzed.

#### Foveal avascular zone (FAZ) area

The OCTA images of all retinal vascular plexuses were exported to ImageJ software (National Institutes of Health, Bethesda, MD, United States). The SCP FAZ area was measured in this study. Manual tracing of the FAZ was done using the “free hand selection” tool of Image J. The FAZ area was manually tracked and measured with “free hand selection” tool of Image J.

#### Perfusion density (PD, %)

The SCP and DCP angiography were then exported to the ImageJ for binarization, described by Ashraf et al. (17) (Figures 1, 2). The binarized images were obtained by using combined adaptive threshold for denoising in Image J, Hessian filter was used to enhance all the microvasculature in the normalized images, and these images were used for quantification of vessel density. To calculate PD, the Image J software extracts a binary image of the blood vessels from the grayscale OCTA image, and then calculates the percentage of the white pixels from the total pixels in the defined region based on the binarized image (Figures 1, 2).

#### Skeleton vessel density (SVD, %)

The SCP and DCP angiography were then exported to the ImageJ for binarization, then, the binary image images were skeletonized with Image J software to generate a vascular tree with a width of 1 pixel, and the total vessel length is calculated described by Ashraf et al. (17) (Figures 1, 2). We duplicated the processed image; one image was processed using a Hessian filter and the other image was processed with a local median threshold. Finally, the two binarized vascular maps were combined to form the final image, and only the pixels common to the two images for the final analysis. The SVD was calculated as a percentage of the skeletonized vessel length divided by the total retinal area based on the skeletonized OCTA image.


$$\mathrm{Skeleton vessel density}=\frac{\mathrm{Total length of skeletonized vessels}}{\mathrm{Total area}-\mathrm{area of}\;\mathrm{FAZ}}$$

### ROC analysis

Receiver operating characteristic (ROC) analysis was used to determine the diagnostic efficacy of the OCTA indicators in distinguishing different stages of DR. The optimal diagnostic threshold of OCTA parameters was determined, that is, the cut-off point with the maximum sum of sensitivity and specificity was selected. These plotted points are connected with lines to form an ROC curve, and the area under the curve (AUC) is defined as the area enclosed by the coordinate axis under the ROC curve. If the AUC is greater than 0.5, it proves that the parameter has certain diagnostic value. At the same time, the closer the area under the ROC curve is to 1, the better the diagnostic value.

### Statistics

All the variables were examined for normality using the Kolmogorov–Smirnov test. The data are presented as the mean ± standard deviation (SD), median and interquartile range (IQR), or percentages as appropriate. The categorical data were assessed using the chi-square test. One-way ANOVA was used to compare the differences among the three groups, and the Bonferroni method was used for *post hoc* tests.

In order to study the changes of various OCTA parameters with the four increased DR severity categories and to explore whether OCTA parameters present a linear trend with the increased DR severity categories, we performed one-way ANOVA combined with *p* trend analysis, and *p* for trend value was calculated. The research factor was DR severity categories (healthy control group, no DR group, NPDR group and PDR group), and the outcome parameters were all the OCTA parameters. All *p*-values were two-sided and considered statistically significant if their value was lower than 0.05. Statistical analysis was performed using the SPSS software version 26 (SPSS, Inc., IL, United States).

## Results

The demographic, ocular, and systemic characteristics of the subjects were shown in Table 1. The study population consisted of 41 healthy control eyes, 25 DM without DR eyes, 27 NPDR eyes, and 23 PDR eyes (Table 1).

**Table 1:** Demographics of healthy and diabetic subjects.

Parameters	Healthy	DM with no DR	NPDR	PDR	*p*-value
Number of eyes, *n*	41	25	27	23	
Age, year	62.78 ± 8.11	60.19 ± 6.69	65.17 ± 7.99	62.98 ± 7.22	*p* = 0.618^a^
Gender, male/female	22/19	13/12	13/14	12/11	*p* = 0.118^b^
Diabetes duration, year	—	5.18 ± 2.25	9.89 ± 3.81	11.64 ± 3.09	**p < 0.001**^a^
HbA1c, %	—	7.47 ± 0.75	8.01 ± 0.87	8.59 ± 0.97	**p < 0.001**^a^
FBG, mmol/L	—	7.40 ± 0.70	7.51 ± 0.99	7.80 ± 1.03	*p* = 0.287^a^
Hypertension prevalence, *n* (%)	4 (9.76%)	3 (12.00%)	3 (11.11%)	2 (8.70%)	*p* = 0.197^b^
Systolic blood pressure, mmHg	122.18 ± 12.14	124.04 ± 7.71	123.21 ± 10.99	124.64 ± 9.05	*p* = 0.385^a^
Diastolic blood pressure, mmHg	79.74 ± 8.11	82.23 ± 8.19	80.37 ± 10.12	83.18 ± 9.44	*p* = 0.528^a^
IOP, mmHg	15.37 ± 1.11	15.02 ± 2.60	14.92 ± 3.13	13.88 ± 3.76	*p* = 0.394^a^
AL, mm	24.21 ± 0.55	23.88 ± 0.69	24.23 ± 0.57	24.03 ± 0.55	*p* = 0.120^a^

AL, axial length; BMI, body mass index; DM, diabetes mellitus; DR, diabetic retinopathy; FPG, fasting blood glucose; PDR, proliferative diabetic retinopathy; NPDR, non-proliferative diabetic retinopathy; IOP, intraocular pressure.

a Statistical significance tested by ANOVA for normal distributions and Kruskal–Wallis tests for nonnormal distributions; all comparisons were corrected with the *post hoc* test.

b *p*-values were calculated using the Fisher’s exact test. Bold values indicate *p* < 0.05.

**Figure 3:**
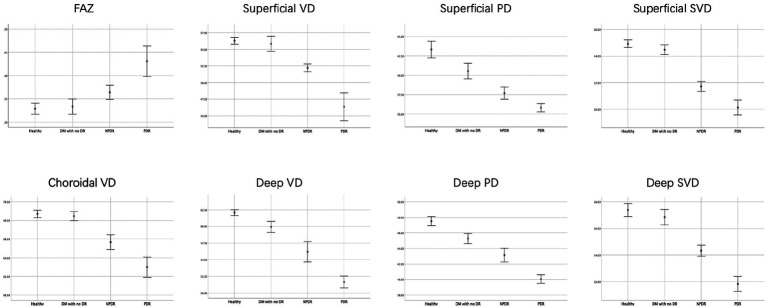
FAZ area, retinal and choroidal vascular density of the four groups. The FAZ area were lower, but mean choroidal VD, the mean retinal parafovea VD, PD and SVD were higher in healthy and DM with no DR eyes compared with NPDR and DR eyes in all vascular layers.

The one-way ANOVAs showed that there is not significantly different in the ONFL thickness, RT, and SFCT among the four groups. There was a significant difference in FAZ area among the 4 groups (healthy, 0.33 ± 0.04 mm^2^; DM without DR, 0.34 ± 0.04 mm^2^; NPDR, 0.36 ± 0.03 mm^2^; PDR, 0.43 ± 0.07 mm^2^; *p* < 0.001, *p* < 0.001) (see Figure 3).

The mean retinal parafovea VD, PD, and SVD were higher in healthy and DM with no DR eyes compared with NPDR and DR eyes in all vascular layers (all *p* < 0.001). The mean choroidal VD were significantly higher in the control group than in the DM, NPDR, and PDR groups (healthy, 68.75 ± 1.20%; DM without DR, 68.45 ± 1.10%; NPDR, 65.80 ± 1.80%; PDR, 63.03 ± 2.19%; *p* < 0.001). The retinal and choroidal vessel density decreased, and FAZ area increased significantly in the two plexuses with worsening DR (see Tables 2, 3).

**Table 2:** Comparisons of the macular parameters in the three groups.

Parameters	Healthy	DM with no DR	NPDR	PDR	*p*-value
Number of eyes, *n*	41	25	27	23	
ONFL thickness, μm	37.15 ± 6.77	36.52 ± 8.12	36.41 ± 4.11	34.13 ± 5.61	*p* = 0.198
Retinal thickness, μm	239.93 ± 20.31	234.64 ± 11.88	239.93 ± 19.21	242.43 ± 15.30	*p* = 0.465
SFCT, μm	212.95 ± 12.24	209.18 ± 9.20	219.33 ± 15.46	228.13 ± 13.70	*p* = 0.201
FAZ area, mm^2^	0.33 ± 0.04	0.34 ± 0.04	0.36 ± 0.03	0.43 ± 0.07	**p < 0.001**
**VD, %**					
SCP	56.35 ± 1.52	55.75 ± 2.41	52.28 ± 1.39	46.92 ± 4.42	**p < 0.001**
DCP	62.07 ± 1.31	59.75 ± 1.82	56.21 ± 3.47	5,171 ± 1.90	**p < 0.001**
**PD, %**					
SCP	43.34 ± 3.39	40.54 ± 2.48	37.68 ± 1.99	35.81 ± 1.28	**p < 0.001**
DCP	47.51 ± 1.92	45.27 ± 1.58	43.14 ± 2.22	40.05 ± 1.27	**p < 0.001**
**SVD, %**					
SCP	14.91 ± 0.87	14.41 ± 0.87	11.82 ± 0.994	10.06 ± 1.14	**p < 0.001**
DCP	17.46 ± 1.46	16.85 ± 1.24	14.30 ± 0.96	11.99 ± 1.20	**p < 0.001**
**Choroidal VD, %**					
Parafovea	68.75 ± 1.20	68.45 ± 1.10	65.80 ± 1.80	63.03 ± 2.19	**p < 0.001**

DCP, deep capillary plexus; DR, diabetic retinopathy; FAZ, foveal avascular zone; PD, perfusion density; PDR, proliferative diabetic retinopathy; ONFL, optic nerve fiber layer; SCP, superficial capillary plexus; SFCT, subfovea choroid thickness; SVD, skeleton vessel density; VD, vessel density.

Bold values indicate *p *< 0.05.

**Table 3:** The *p* trend analysis was performed between OCTA parameters and the DR severity categories.

OCTA parameters	*p* trend analysis	
	*F*	*p* for trend
SCP FAZ area, mm^2^	74.379	**<0.001**
VD, %		
SCP	230.243	**<0.001**
DCP	360.423	**<0.001**
PD, %		
SCP	141.181	**<0.001**
DCP	264.016	**<0.001**
SVD, %		
SCP	478.696	**<0.001**
DCP	328.805	**<0.001**
Choroidal VD, %	232.250	**<0.001**

DCP, deep capillary plexus; DR, diabetic retinopathy; FAZ, foveal avascular zone; PD, perfusion density; SCP, superficial capillary plexus; SFCT, subfovea choroid thickness; SVD, skeleton vessel density; VD, vessel density.

Bold values indicate *p *< 0.05.

All the OCTA parameters show a significant difference across disease groups in *p* trend analysis. FAZ area was inversely associated with DR severity categories (*p* for trend <0.001). Different stages of DR were significantly associated with VD, PD, and SVD of SCP (all *p* for trend <0.001). For DCP, there are also significant correlations among VD, PD, SVD, and DR severity categories (*F* = 360.423; *p* for trend <0.001; *F* = 264.016, *p* for trend <0.001; *F* = 328.805, *p* for trend <0.001; respectively). Decreasing Choroidal VD values were associated with improved DR severity (*p* for trend <0.001).

**Figure 4:**
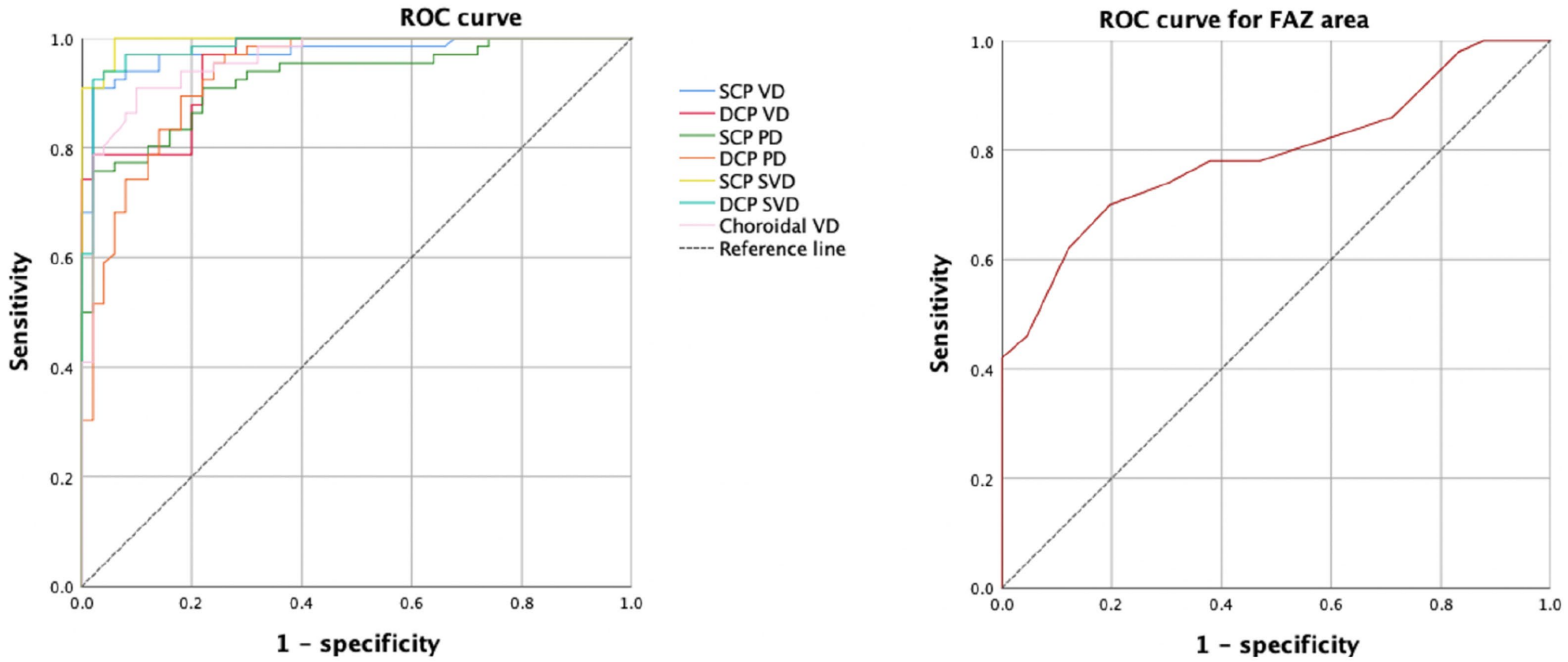
The ROC curve for FAZ area, the retinal and choroidal vessel density.

The ROC curve for FAZ area, the retinal and choroidal vessel density were analyzed (Figure 4). For the FAZ area, the AUC was 0.792.

Both the SCP and DCP vessel density showed the ability to distinguish healthy eyes from eyes with DR. The AUCs of 0.971 for SCP vessel density and 0.956 for DCP vessel density. The AUCs of skeleton vessel density showed 0.997 for superficial layer and 0.980 for deep layer. For the choroidal vessel density, the AUC was 0.956. The AUCs of perfusion density showed 0.923 for superficial layer and 0.932 for deep layer.

## Discussion

The previous studies have used OCT and OCTA to evaluate the characteristics of different retinal/choroidal vascular plexuses capillary perfusion in DR patients. In our previous research, we found that the choroidal blood flow decreased with severity of DR eyes compared with DM and normal eyes, and these changes may predict DR development before they are otherwise evident clinically (18). However, we did not analyze the retinal blood flow density, nor did we explore the diagnostic value of each parameter for DR severity in my previous research. In this study, we evaluated the changes in fundus vascular density and micromorphological structure of all vascular plexuses during the different stages of diabetic retinopathy (DR), and the correlation between fundus blood flow and the DR severity. The ROC curve analysis was used to compare the diagnostic value of different blood perfusion parameters for DR. The current study demonstrated that the density and morphological features of retinal blood vessels can be quantified by OCTA to assist in assessing the severity of DR.

Previous studies have shown that diabetic eyes had significantly enlarged FAZ area compared to healthy controls (8, 19–23). However, for DR patients, there are some mixed findings about whether FAZ is greater in patients with PDR or NPDR. Some studies reported that they did not find a significant FAZ area difference between NPDR and PDR eyes (20) while others found significantly enlarged FAZ area in PDR eyes compared to NPDR eyes (15, 24). In this study, we found that the FAZ area of NPDR and PDR eyes was significantly larger compared to the eyes of the normal control group, and the increase in FAZ in PDR eyes was more significant.

It has been confirmed that retinal VD correlates with the severity of DR, and it has become a new indicator of fundus hemodynamic changes and widely used in clinical research recently (5–7). In the study of Onishi et al. (9), VD was significantly reduced in all three plexuses in DR patients compared to healthy controls. Dimitrova et al. (25) showed a significantly decrease of superficial and deep retinal vessel density in diabetic patients compared to healthy people. This study confirmed that with the exacerbation of DR, the density of capillary perfusion at all levels of the retina decreased significantly, which is consistent with previous results (9, 14). Wang et al. (26) performed a quantitative analysis of retinal microvasculature in patients with early-stage DR using wide-field OCTA and found that the VD of the no DR and mild-moderate NPDR were significantly decreased in the 3 mm radius compared with the control group in superior and inferior quadrants. Yuan et al. (12) explored the associations of peripapillary microvascular metrics with DR incidence and development using swept-source OCTA (SS-OCTA), lower peripapillary VD and peripapillary vessel length density (VLD) of SCP are associated with 2 years incident DR among the type 2 diabetes mellitus population. These early changes in retinal blood flow density demonstrating that the blood flow density change can be used to distinguish between normal eyes and DR eyes and can distinguish the severity of DR, and that changes in retinal capillaries in diabetic patients might occur earlier than clinical symptoms.

In order to explore whether the decreased retinal blood perfusion in patients with diabetic retinopathy occurs in large vessels or capillaries, Lei et al. (27) evaluated the blood flow status of superficial capillaries and large vessels separately and concluded that blood flow in retinal capillaries and large vessels did not respond consistently in diabetic retinopathy and decreased retinal hemoperfusion is mainly manifested in the capillary layer. In recent years, there have also been some studies on changes in retinal blood vessel diameter of DR eyes. Tang et al. (28) recently reported that retinal vascular diameter increases with worsening DR, suggesting telangiectasia and hyperperfusion were exist in DR eyes, and the parameter describing the diameter of blood vessels is called the retinal diameter index. Lei et al. (27) found that as DR progressed, the diameter of large blood vessels in the retina gradually increased, which may be due to dilation of small retinal venules or increased retinal blood flow during DR. These results are consistent with previous studies of retinal blood flow in diabetic retinopathy patients using Doppler velocimetry (29, 30).

At the same time, many researchers believe that blood flow density various in different stages of the progression of diabetic retinopathy are not completely consistent. Onishi et al. (9) found the adjusted flow index (AFI), a parameter that approximates blood flow, increased in DM with no DR eyes compared with normal control eyes. But when it progresses to the stage of diabetic retinopathy, SCP, MCP and DCP AFI showed a decreasing trend with the aggravation of diabetic retinopathy, and the decreasing trend of MCP and DCP AFI was more obvious. One possible explanation is that there may be automatic regulation of SCP blood flow in the early stage of DR, the superficial retinal blood flow remains stable, but the deep retinal blood flow may steeply decrease. The increase in retinal blood supply in the early stages of DR is consistent with some previous studies (31, 32). However, the other studies showed that retinal blood flow in the early stage of DR was not increased, and with progression of retinopathy, retinal blood flow is reduced at first and gradually increased in the later stage (30). However, most mainstream studies still believe that in early stage of DR, SCP parameters were significantly altered compared to healthy eyes, and with the increase in the severity of DR, the changes in the deep plexus are more obvious (9, 14).

Recent studies explored a new variety of other OCTA parameters, mainly including some parameters analyzed after binary processing of OCTA image, such as vessel length density (VLD), perfusion density (PD), skeletonized vessel density (SVD), fractal dimensions (FD), vessel diameter index (VDI) and adjusted flow index. In the study of Kim et al. (6), significantly lower SVD, VD, FD, and higher VDI were found in NPDR subjects than the healthy controls. Agemy et al. (7) reported a significant reduction in capillary PD in almost all layers in all study groups with controls.

Ashraf et al. (24) identified a set of OCTA parameters, such as the SCP FAZ area, DCP vessel density, and acircularity with high sensitivity and specificity for distinguishing DR severity. Lei et al. (27) proposed capillary vessel length density (VLD) would be a potentially reliable parameter for evaluation of DR, it has a high sensitivity and specificity in detecting different stages of DR. VLD is actually a similar concept to SVD, in the present study, the AUC of skeleton vessel density is higher than vessel density, which was consistent with previous study. Previous studies also reported that skeleton density (SD) is a more accurate measure of retinal vascular changes because the skeletonized image normalizes the diameter of larger vessels with that of capillaries, removing the influence of vessel size on retinal perfusion measurements (6, 10). We analyzed the possible reasons why the diagnostic value of SVD was higher than that of VD, VD is obtained by the built-in AngioVue Analytics software, and the presence of projection artifacts from the OCTA images of the vascular plexus were existed. The existence of these artifacts will also make the analysis results less accurate, but for the measurement of SVD, the angiography was binarized and skeletonized to generate a vascular tree with a width of 1 pixel, the total vessel length is calculated. The SVD was calculated as a percentage of the skeletonized vessel length divided by the total retinal area. Therefore, this parameter can remove the influence caused by image artifacts and better reflect the vascular density, which has higher diagnostic value. The results of different stratified vascular parameters of retina on the diagnosis and evaluation of the severity of diabetic retinopathy are also inconsistent. Durbin et al. (8) reported the ability to distinguish healthy eyes from eyes with DR, with AUCs of 0.893 for VD and 0.794 for PD in the superficial retinal layer, indicating that the diagnostic value for superficial retinal vascular density is higher than that of deep retinal vascular density. While other researchers believed that DCP parameters as more important (33). In the present study, the mean vascular density of all vascular plexuses had a high AUC, suggesting that these OCTA parameters showed a high diagnostic value, a high sensitivity and specificity for DR.

This study also has certain limitations. First, we included a relatively small number of eyes, so it is difficult to draw definitive conclusions based on this study alone, and larger sample sizes are needed to verify and draw scientific conclusions. Second, the automatic segmentation algorithm used in this study is also prone to segmentation errors, which have a certain impact on the result. The previous research has analyzed the blood flow in three retinal layers, and changes in the subtle structure of the retina can be explored in greater detail. However, due to the lack of software and algorithm, we can only analyze the superficial and deep retinal blood vessels with the built-in AngioVue Analytics software. Another limitation is that although the OCTA image binarization technique has been widely used in clinical research, different thresholding techniques may significantly affect the measurements.

In conclusion, the OCTA data can be used to quantify the severity of diabetic retinopathy and provide early indications before fundus lesions occur in diabetic patient. The decreasing vessel density in the SCP and DCP were associated with worsening DR. We also present OCTA parameters from the SCP and DCP to achieve high sensitivity and specificity for distinguishing DR and healthy eyes. The retinal and choroidal vascular parameters might be potential early biomarkers indicating the progression of DR before appearance of clinically PDR in patients with DM with high sensitivity and specificity.

## Data availability statement

The raw data supporting the conclusions of this article will be made available by the authors, without undue reservation.

## Ethics statement

The studies involving human participants were reviewed and approved by the Ethics Committee of Beijing Chaoyang Hospital, Capital Medical University. The patients/participants provided their written informed consent to participate in this study. Written informed consent was obtained from the individual(s) for the publication of any potentially identifiable images or data included in this article.

## Author contributions

HW and SY: involved in the design of the study and critical revision of the manuscript. HW and XH: conduct the study. HW, XL, and HB: collection, patients’ epidemiological survey, and baseline data statistics. XL and XH: patient follow-up data collection. HW: preparation of the manuscript. All authors contributed to the article and approved the submitted version.

## Funding

This work was supported by Science and Technology Innovation Foundation of Beijing Chaoyang Hospital (no. 22kcjjyb-14); National Natural Science Foundation of China (no. 62006161). These fundings had no role in study design, data collection and analysis, decision to publish, or preparation of the manuscript.

## Conflict of interest

The authors declare that the research was conducted in the absence of any commercial or financial relationships that could be construed as a potential conflict of interest.

## Publisher’s note

All claims expressed in this article are solely those of the authors and do not necessarily represent those of their affiliated organizations, or those of the publisher, the editors and the reviewers. Any product that may be evaluated in this article, or claim that may be made by its manufacturer, is not guaranteed or endorsed by the publisher.
